# Derivation of Porcine Extra-Embryonic Endoderm Cell Lines Reveals Distinct Signaling Pathway and Multipotency States

**DOI:** 10.3390/ijms222312918

**Published:** 2021-11-29

**Authors:** Man-Ling Zhang, Yong Jin, Li-Hua Zhao, Jia Zhang, Meng Zhou, Mei-Shuang Li, Zhi-Bao Yin, Zi-Xin Wang, Li-Xia Zhao, Xi-He Li, Rong-Feng Li

**Affiliations:** 1 The State Key Laboratory of Reproductive Regulation and Breeding of Grassland Livestock, College of Life Science, Inner Mongolia University, Hohhot 010020, China; zhangmanling@mail.imu.edu.cn (M.-L.Z.); 21908026@mail.imu.edu.cn (J.Z.); zhaolixia0814@163.com (L.-X.Z.); 2 Jiangsu Key Laboratory of Xenotransplantation, Nanjing Medical University, Nanjing 211166, China; jinyong@njmu.edu.cn (Y.J.); zhaolihua@njmu.edu.cn (L.-H.Z.); mengzh10@njmu.edu.cn (M.Z.); meishuang@njmu.edu.cn (M.-S.L.); yinzhibao@njmu.edu.cn (Z.-B.Y.); 3 Key Laboratory of Targeted Intervention of Cardiovascular Disease, Collaborative Innovation Center for Cardiovascular Disease Translational Medicine, Nanjing Medical University, Nanjing 211166, China; 4 Inner Mongolia Saikexing Institute of Breeding and Reproductive Biotechnology in Domestic Animal, Hohhot 011517, China; wangzixin123456@163.com

**Keywords:** porcine blastocysts, extra-embryonic endoderm, signaling pathway, stem cells

## Abstract

The inner cell mass of the pre-implantation blastocyst consists of the epiblast and hypoblast from which embryonic stem cells (ESCs) and extra-embryonic endoderm (XEN) stem cells, respectively, can be derived. Importantly, each stem cell type retains the defining properties and lineage restriction of its in vivo tissue origin. We have developed a novel approach for deriving porcine XEN (pXEN) cells via culturing the blastocysts with a chemical cocktail culture system. The pXEN cells were positive for XEN markers, including Gata4, Gata6, Sox17, and Sall4, but not for pluripotent markers Oct4, Sox2, and Nanog. The pXEN cells also retained the ability to undergo visceral endoderm (VE) and parietal endoderm (PE) differentiation in vitro. The maintenance of pXEN required FGF/MEK+TGFβ signaling pathways. The pXEN cells showed a stable phenotype through more than 50 passages in culture and could be established repeatedly from blastocysts or converted from the naïve-like ESCs established in our lab. These cells provide a new tool for exploring the pathways of porcine embryo development and differentiation and providing further reference to the establishment of porcine ESCs with potency of germline chimerism and gamete development.

## 1. Introduction

Embryogenesis is usually accompanied by a gradual loss of the developmental ability of the totipotent zygote. After a series of cleavage and early differentiation of the blastomeres, the embryo develops into the blastocyst stage that is characterized by the presence of a fluid-filled cavity and an inner cell mass (ICM), which are together surrounded by the trophectoderm (TE) [1]. The ICM cells exposed to the fluid cavity develop into the hypoblast (primitive endoderm, PrE), while the remaining cells surrounded by the TE and PrE become the epiblast (EPI) [2]. The TE cells contribute to the placenta formation; the EPI cells are the source of most of the embryo proper, the amnion, and the extra-embryonic mesoderm of the yolk sac; and the PrE cells form the two extra-embryonic endoderm (ExEn) lineages, the visceral endoderm (VE) and parietal endoderm (PE) of the yolk sac [3]. Therefore, stem cell lines can be established from three tissue lineages present in the blastocyst and are used as an in vitro model to study the regulation of early lineage progenitors.

Embryonic stem cells (ESCs) are derived from the ICM of pre-implantation blastocysts [4,5], and express the pluripotency associated transcription factors Oct4, Sox2, and Nanog [6,7,8]. Trophoblast stem cells (TSCs) are derived from the TE cells; the TSCs resemble gastrulation-stage trophectoderm and express the specific marker of Cdx2 [9]. Extra-embryonic endoderm (XEN) cells are derived from the PrE and resemble the parietal endoderm; the XEN cells express Gata4, Gata6, and Sox17 [10]. All three types of stem cells are self-renewing in vitro, but they differentiate and contribute to appropriate lineage patterns both in vitro and in vivo.

At present, authentic ESC lines and optimized in vitro culture systems have not been established for pigs, although pigs are potential candidates for human disease models because of their similar size and physiology to humans [11]. Many researchers have attempted to establish ESCs from porcine embryos, but the obtained cells only have characteristics of pluripotent stem cells [12,13,14]. Our lab has also been devoted to the research on the derivation of porcine ESCs [15,16], but producing authentic ESCs remains a challenge. Yang et al. showed that a chemical cocktail enabled the derivation of extended pluripotent stem cells from mice and humans and that the derived cells were capable of forming chimeras of both embryonic and extra-embryonic tissues [17]. Subsequently, Gao et al. reported the derivation of expanded potential stem cells from pigs by inhibiting the critical molecular pathways that drive lineage differentiation in vitro [18]. Based on these researches, we tried to cultivate porcine blastocysts using Yang’s culture system; however, surprisingly, we obtained cells with a flat morphology that did not express ESCs pluripotent markers, but instead showed typical characteristics of XEN cells.

In mice, XEN cells can be obtained in three ways: by directly derivation from blastocysts [19]; by conversion of ESCs through the expression of transcription factors [20,21,22], or through chemical modification of the culture medium [23,24]; and by parallel generation of induced XEN during induced pluripotent stem (iPS) cell production [25]. Isolation of porcine XEN cells has rarely been reported; only two groups have reported deriving XEN cells from porcine blastocysts [26,27], and one group has derived XEN cells from porcine pluripotent stem cells [28]. These researchers obtained XEN cells by adding Fibroblast Growth Factor (FGF) and Leukemia Inhibitory Factor (LIF) to the culture medium, but made little mention of the specific signaling pathways that XEN cells rely on for self-renewal or the differences between XEN cells and different states of porcine ESCs.

In the present study, we used a chemical cocktail culture system to generate porcine XEN (pXEN) cells from day 6 blastocysts and naïve-like porcine ESCs (nESCs). The pXEN cells could be cultured stably, expressed XEN marker genes and differentiated into VE and PE. The maintenance of pXEN cells depended on the Mitogen Extracellular signal-regulated kinase/extracellular signal-regulated kinase (MEK/ERK) and transforming growth factor-β (TGF-β) signaling pathways, and their gene expression pattern differed significantly from that of porcine naïve-like and primed-state of pluripotent stem cells. The pXEN cells maintained a stable phenotype through over 50 passages in culture; therefore, they can serve as useful tools for exploring the pathways of porcine embryo development and differentiation.

## 2. Results

### 2.1. Derivation of Porcine XEN Cell Lines from Blastocysts

**Figure 1:**
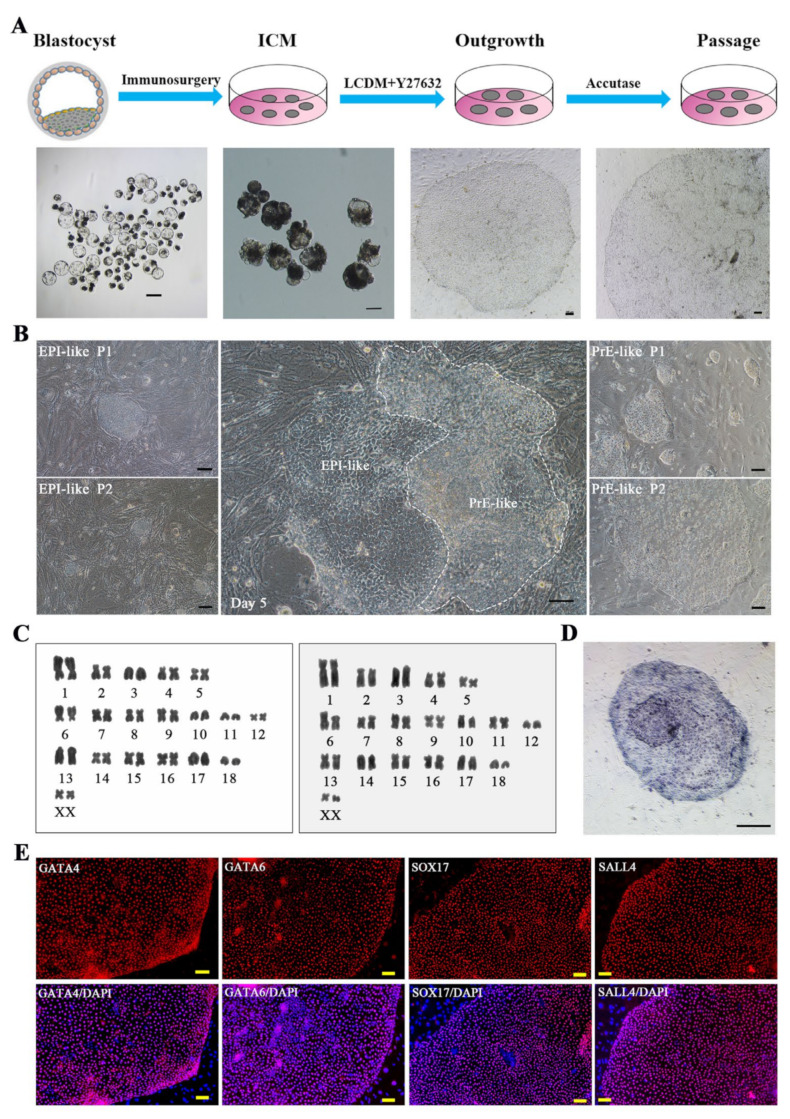
Derivation and characterization of pXEN cells. (**A**) Schematic diagram of establishment of pXEN cells from inner cell masses (ICMs) of day 6 in vitro parthenogenetic porcine embryos. (**B**) Image of outgrowth formed from ICMs contained two morphologic cell types and subsequent generations of these two cell types. (**C**) Karyotype analysis of pXEN cell line 1 at passage 29 and cell line 2 at passage27. (**D**) AP staining of pXEN cells at passage 12. (**E**) The immunofluorescence staining of XEN markers GATA4, GATA6, SOX17, and SALL4 in pXEN cell line 1 colonies (at passage 21) cultured on mouse embryonic fibroblast (MEF) cells. Scale bar, 100μm.

**Figure 2:**
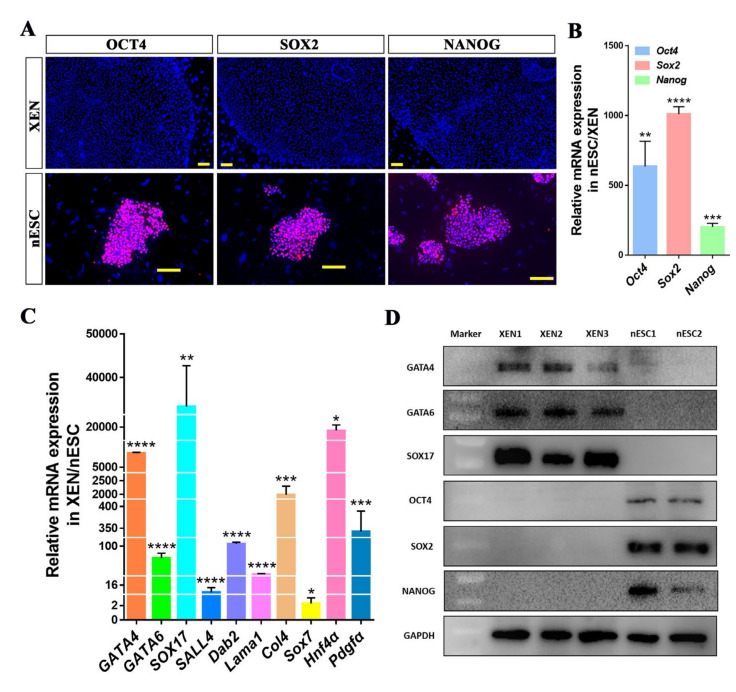
Characteristics of pXEN cells. (**A**) The immunofluorescence staining of pluripotency markers OCT4, SOX2, and NANOG in pXEN cell line 1 colonies (the cells were cultured to 21 passages) and porcine naïve-like ESCs (the cells were cultured to 19 passages) cultured on MEF cells. (**B**) Quantitative RT-PCR analysis for expression levels of Oct4, Sox2, and Nanog in pXEN cells and porcine nESCs. Relative expression reflected as a fold difference in porcine nESCs compared to pXEN cells, pXEN cells = 1. (**C**) Quantitative RT-PCR analysis for expression levels of XEN cell markers in pXEN cells and porcine nESCs. Relative expression reflected as a fold difference in pXEN cells compare to porcine nESCs, porcine nESCs =1. Data are depicted as mean ± SD. *p < 0.05, **p < 0.01, ***p < 0.001, ****p < 0.0001 versus Control (*t*-test). (**D**) Western blotting analysis confirming the expression of XEN cell markers (GATA4, GATA6, and SOX17) in pXEN cells, and the expression of pluripotent markers (OCT4, SOX2, and NANOG) in porcine nESCs. Scale bar, 100μm.

The ICMs derived from Day 6 porcine blastocysts were cultured in LCDM (LIF, CHIR99021, (S)-(+)-dimethindene maleate, and minocycline hydrochloride were added into the basal medium) medium, which enabled the derivation of extended pluripotent stem cells previously [17], and 3–5 days later, the outgrowth appeared (Figure 1A). Some colonies formed from ICMs contained two morphologic cell types: one type showed uniform and dense cell morphology (EPI-like), and the other type showed loose and epithelial cell morphology (PrE-like) and were adjacent to the EPI-like cells. We tried to separate these two types of cells and subculture them separately during this period; however, the EPI-like cells differentiated after 2–3 generations of in vitro culture and the clones could not continue to grow, while the PrE-like cells were more stable in the subsequent culture processes (Figure 1B). Most colonies formed directly from the PrE-like cells and had a large and flat morphology after 10–12 days of culture. The enlarged outgrowth was mechanically isolated and seeded on fresh feeder cells. These cells could be mechanically passaged or digested with accutase into clumps for passage but did not survive after single cell digestion. The addition of Y27632 increased the colony formation rate (Supplementary Figure S1). The cells displayed a continued normal 38XX karyotype at different passages (Figure 1C) and were alkaline phosphatase (AP) positive (Figure 1D). The immunofluorescence and real-time PCR analyses showed that these cells were positive for XEN markers, including Gata4, Gata6, Sox17, and Sall4 (Figure 1E and Figure 2C), but not for the pluripotent markers Oct4, Sox2, and Nanog (Figure 2A,B). The expression of XEN markers, such as *Dab2*, *Lama1*, *Col4*, *Sox7*, *Hnf4α* and *Pdgfra*, was also at significantly higher levels in these cells than in porcine nESCs (Figure 2C). Western blotting analysis further confirmed that the cells expressed XEN markers GATA4, GATA6, and SOX17, but not pluripotent markers OCT-4, SOX2, and NANOG (Figure 2D). Therefore, we gave the stably passaged cells the name porcine XEN (pXEN) cells. We obtained three stable cell lines, and they could be maintained for at least 50 passages in vitro.

### 2.2. In Vitro Differentiation Potential of pXEN Cells

**Figure 3:**
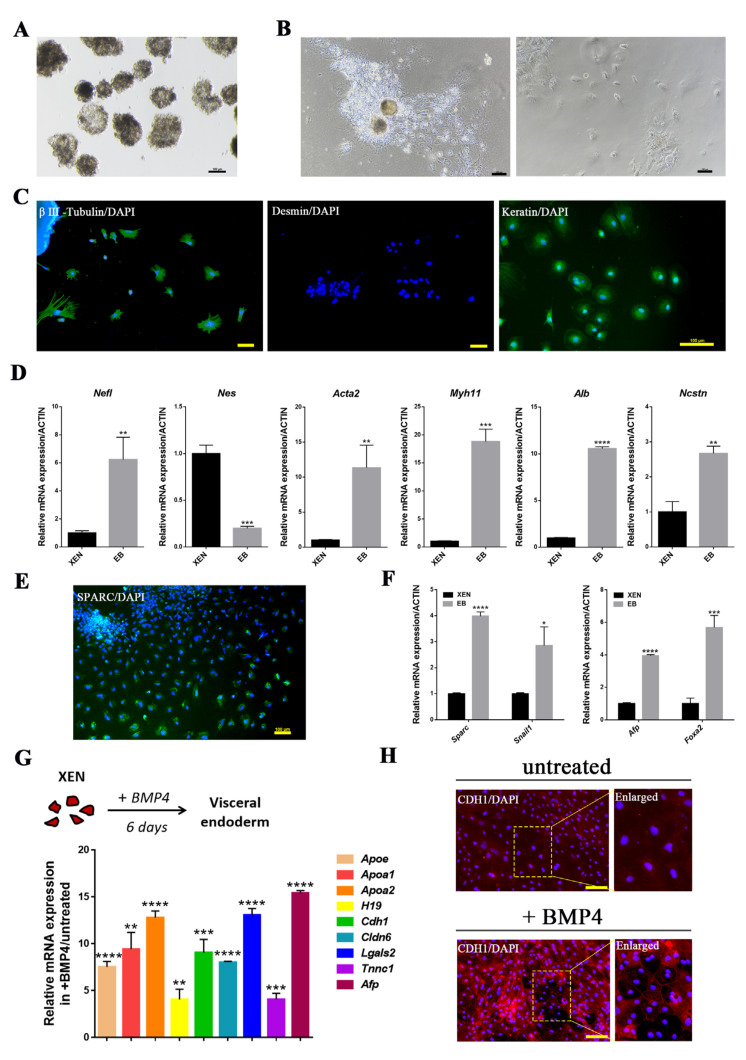
In vitro differentiation potential of pXEN cells. (**A**) Embryoid body (EB)-like spheroids derived from pXEN cell line 1 at passage 23. (**B**) EB-like spheroids spread on dishes coated with gelatin displayed distinct signs of differentiation. (**C**) Expression of differentiation markers βIII-Tubulin, Desmin, and Cytokeratin from differentiated pXEN cells were detected by immunocytochemistry analysis. (**D**) Quantitative RT-PCR for the expression of differentiation related genes in EB-like spheroids and pXEN cells. Data are depicted as mean ± SD. * *p* < 0.05, ** *p* < 0.01, *** *p* < 0.001, **** *p* < 0.0001 versus Control (*t*-test). (**E**) The immunofluorescence staining of the PE marker Sparc in differentiated pXEN cells. (**F**) Quantitative RT-PCR for the expression of VE (Afp, foxa2) and PE (Sparc, Snail1) markers in EB-like spheroids and pXEN cells. (**G**) VE differentiation assay and quantitative RT-PCR analysis of VE gene expression in differentiated pXEN cells and untreated cells. Relative expression reflected as a fold difference in differentiated pXEN cells compared to untreated cells, untreated cells = 1. (**H**) Immunofluorescence shows expression of the VE marker CDH1 is significantly higher at cell junctions in differentiated pXEN cells than in untreated cells. Scale bar, 100μm.

The classic approach to inducing ESC differentiation is to allow ESCs to grow in suspension and form Embryoid bodies (EBs). Within the EBs, the process of ESC differentiation is similar to that in embryos. The differentiation ability of XEN cells was also determined here by EB formation assays, and we found that the pXEN cells could form EB-like spheroids when grown in suspension without LIF, CHIR99021, (S)-(+)-dimethindene maleate, and minocycline hydrochloride for 7 days (Figure 3A). Day-5 EB-like spheroids were plated onto gelatin-coated dishes and grown in basal medium without LIF, CHIR99021, (S)-(+)-dimethindene maleate, and minocycline hydrochloride and the cells in the outgrowths of the EB-like spheroids displayed the morphology of distinct differentiation (Figure 3B). Immunofluorescence analysis confirmed the expression of differentiation markers βIII-Tubulin and Cytokeratin in these differentiated cells, but the expression of Desmin was negative (Figure 3C). Real-time PCR analyses showed that the expressions of the differentiation related genes *Nefl*, *Acta2*, *Myh11*, *Alb* and *Ncstn* were all significantly increased in the EB-like spheroids compared with undifferentiated cells, but the expression of *Nestin* was not increased (Figure 3D). The pXEN cells derived in our lab did not achieve the pluripotent state, because no mesoderm cells formation during EB differentiation, which was consistent with previous study on porcine XEN cells [28]. The expression of ectoderm-related gene *Nestin* was not up-regulated, indicating that the pluripotency of pXEN cells was lower than that of pluripotent stem cells, and the potential of XEN cells to differentiate in vitro was limited to some extent.

Since previous studies showed that blastocyst-derived XEN cells can differentiate into VE or PE [10,29], we evaluated the multipotency of our pXEN cells. We detected the expression of the PE marker Sparc in the differentiated cells in EB-like spheroids (Figure 3E), and the expression of the PE marker genes *Sparc* and *Snail*, and VE marker genes *Afp* and *Foxa2* were upregulated in the differentiated cells (Figure 3F). Some studies have shown that PE is an intermediate state that will eventually be reprogrammed to VE; therefore, we used BMP4 to treat the cells in VE differentiation assays, and we found that pXEN cells had differentiated to VE six days later, as evidenced by epithelialization, increased localization of E-cadherin (CDH1) at cell boundaries (Figure 3H), and the upregulation of VE markers, such as *Apoe*, *Apoa1*, *Lgals2*, and *Afp* (Figure 3G).Based on these, we concluded that the pXEN cells had the ability to differentiate into both VE and PE.

### 2.3. Maintenance of pXEN Cells Was Dependent on FGF/MEK and TGFβ Signaling

**Figure 4:**
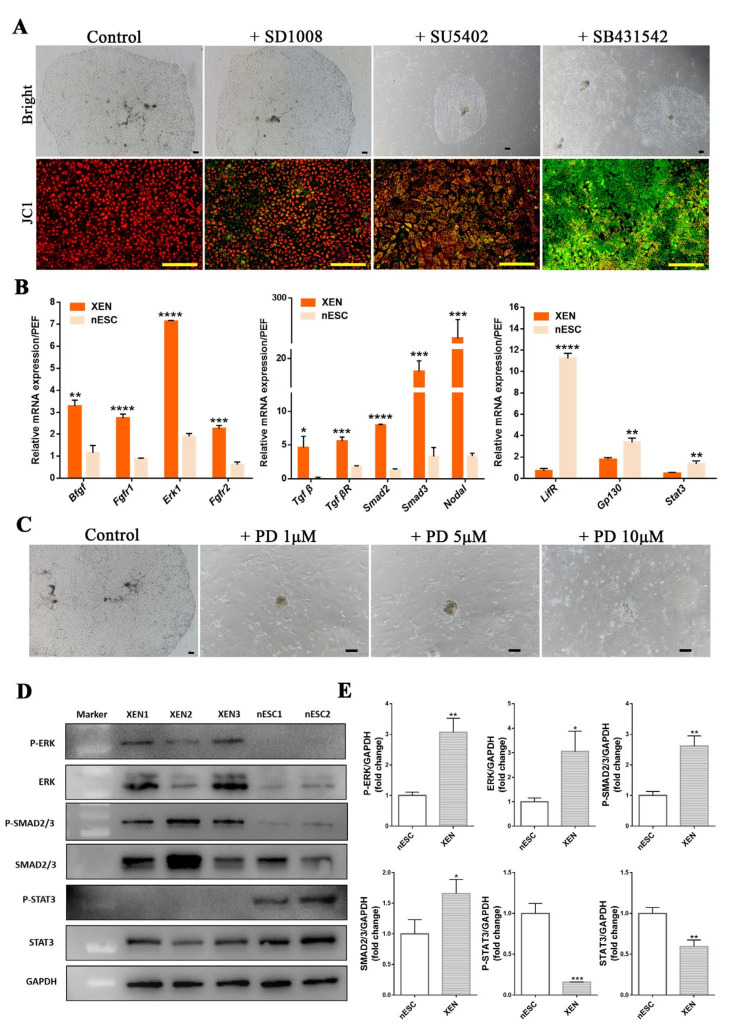
Signaling dependence analysis of pXEN cells. (**A**) The morphology and JC1 staining of pXEN cells cultured in LCDM, LCDM+10μm SD1008, LCDM+10μm SU5402 and LCDM+10μm SB431542. (**B**) Quantitative RT-PCR analysis of FGF, TGFβ and LIF signaling related genes in pXEN cells, porcine nESCs and porcine embryo fibroblasts (PEF). Relative expression reflected as a fold difference in pXEN cells and porcine nESCs compared to PEF, PEF = 1. Data are depicted as mean ± SD. * *p* < 0.05, ** *p* < 0.01, *** *p* < 0.001, **** *p* < 0.0001 versus Control (*t*-test). (**C**) The morphology of pXEN cells cultured in LCDM and LCDM supplemented with 1, 5 and 10μM concentration of PD0325901. (**D**) Western blotting analysis of the phosphorylation status of ERK, STAT3 andSMAD2/3, and the expression of ERK, STAT3 andSMAD2/3 in pXEN cells and porcine nESCs. (**E**) The quantification of proteins after Western blotting. Scale bar, 100μm.

Some studies have suggested that the fibroblast growth factor (FGF) receptor and the TGFβ family member Nodal are expressed in PrE [30,31]; therefore, we examined whether pXEN cells could proliferate and grow in the presence of the FGF receptor inhibitor SU5402, the LIF signaling inhibitor SD1008, or the TGFβ receptor inhibitor SB431542. We plated pXEN cells in LCDM media treated with SU5402, SD1008, or SB431542 and evaluated the cell proliferation and viability by performing a JC1 (5,5′,6,6′-tetrachloro-1,1′,3,3′-tetraethylbenzimi-dazolylcarbocyanine iodide, cyanine dye) assay at 5 days after the inhibitor treatment. After staining cells with JC1, the ratio of red fluorescence (corresponding to activated mitochondria; J-aggregates) to green fluorescence (corresponding to less active mitochondria; J-monomers) can be calculated to detect mitochondrial membrane potential, which is an important parameter of mitochondrial function and can be used as an indicator of cell health. The results showed that LIF signaling inhibitor had little effect on cell proliferation and viability, even at 10 μM concentration, because the cells emitted high red fluorescence indicated a high mitochondrial membrane potential. Surprisingly, SU5402 restricted cell proliferation to some extent, while the presence of SB431542 led to loss of mitochondrial membrane potential in the cells that emitted high green fluorescence, which indicated the decreased viability of cells (Figure 4A). Real-time PCR analysis showed upregulated expression of genes involved in FGF and TGFβ signaling pathways and downregulated expression of LIF signaling pathway-related genes in pXEN cells (Figure 4B). To verify the effect of MEK-ERK signaling, downstream of FGF pathway on the maintenance of pXEN cells, we cultured the pXEN cells in LCDM and LCDM supplemented with 1, 5 and 10 μM concentrations of the ERK signaling inhibitor PD0325901. The results showed that the viability of pXEN cells was affected when PD0325901 was added to LCDM even at a concentration of 1μM, and the cells could not proliferate (Figure 4C). Although SU5402 could compromise the proliferation of pXEN cells to some extent, it will not prevent the proliferation of cells, while PD0325901 can prevent the normal proliferation of cells, suggesting that the effect of cells treated with SU5402 may not be equivalent to PD0325901. Western blotting showed that phosphorylation of STAT3 was detected in nESCs but not in pXEN cells, whereas phosphorylation of ERK occurred in pXEN cells but not in nESCs. Smad2/3 and phosphorylated Smad2/3 protein were both highly expressed in pXEN cells (Figure 4D). The quantification of proteins after Western blotting were consistent with these results (Figure 4E), further confirming that the LIF pathway was not required to maintain pXEN cells, whereas the maintenance of pXEN cells required the FGF/MEK and TGFβ signaling pathways.

### 2.4. Conversion of Porcine Naïve-like ESCs to XEN Cells

**Figure 5:**
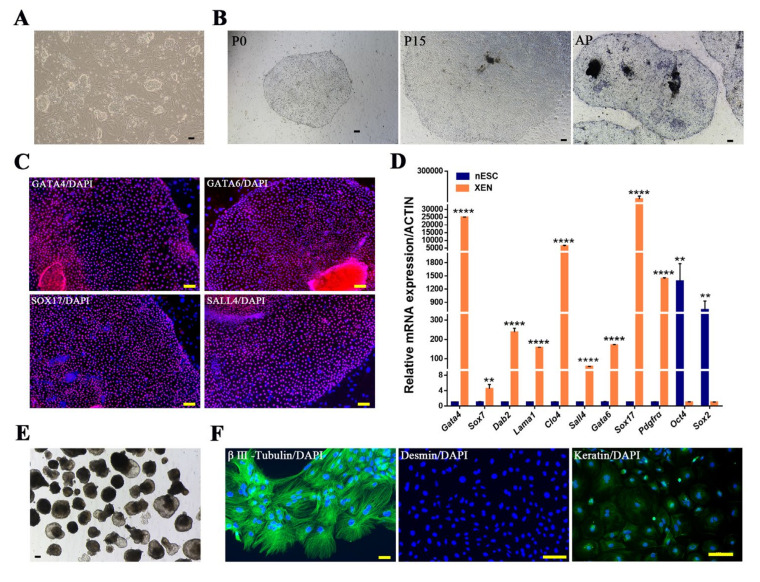
Conversion of porcine nESCs to XEN cells. (**A**) The morphology of nESCs colony at passages 19. (**B**) Porcine nESCs were cultured in LCDM, and the colonies converted to pXEN morphology after 7 days (P0); these cells could be passaged through accutase (P15) and were positive for AP staining. (**C**) The immunofluorescence staining of XEN markers GATA4, GATA6, SOX17, and SALL4 in the converted pXEN cells. (**D**) Quantitative RT-PCR analysis for expression levels of XEN and pluripotency markers in converted pXEN cells and porcine nESCs. Data are depicted as mean ± SD. ** *p* < 0.01, **** *p* <0.0001 versus Control (*t*-test). (**E**) Embryoid body (EB)-like spheroids derived from converted pXEN cells. (**F**) Immunofluorescence analysis of the expression of differentiation markers from differentiated converted pXEN cells. Scale bar, 100μm.

Studies have shown that ESCs can be converted into XEN cells [24]. In our lab, we had established porcine naïve-like ESCs (nESCs) that showed characteristics was very similar to those of mouse ESCs, as they depended on LIF signal to maintain pluripotency, could differentiate into three germ layers, and could be induced to undergo directional differentiation under defined conditions in vitro [16]. Therefore, we cultured porcine naïve-like ESCs (Figure 5A) in LCDM medium and found distinct colonies with morphological similarities to XEN cells after 7 days. Colonies derived from these porcine nESCs could be expanded for at least 40 passages and were AP positive (Figure 5B). We next asked whether converted XEN cells were molecularly identical to embryo-derived XEN cells by performing immunofluorescence analysis for XEN markers. The converted XEN cells showed expression of Gata4, Gata6, Sox17, and Sall4 (Figure 5C). Real-time PCR analyses confirmed these cells expressed XEN associated genes, such as *Gata4, Gata6, Sox17, Pdgfra, Col4* and *Dab2*, at significantly higher levels than was observed in nESCs, but the expression of pluripotent markers including *Oct4* and *Sox2*, was significantly lower in the converted XEN cells than in nESCs (Figure 5D). The converted XEN cells could also form EB-like spheroids (Figure 5E). Immunofluorescence analysis confirmed the expression of the differentiation markers βIII-tubulin and cytokeratin in the differentiated cells, and the expression of desmin was still negative (Figure 5F).

### 2.5. Comparison of pXEN Cells and Primed ESCs

**Figure 6:**
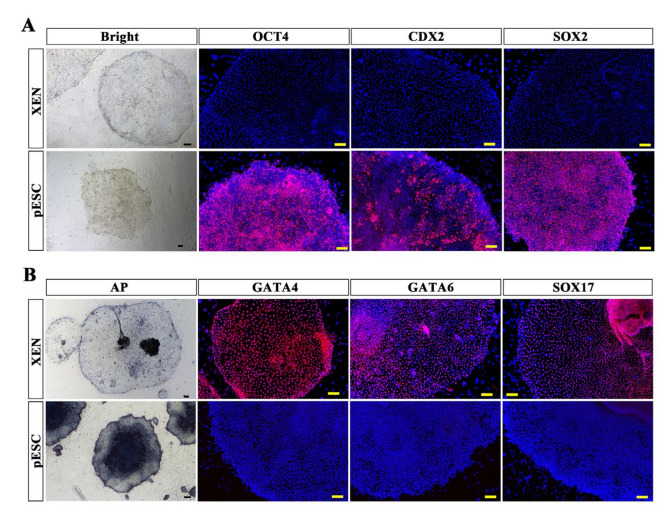
Comparison of pXEN cells and porcine primed ESCs (pESCs). (**A**) Bright field photographs and immunofluorescence staining of pluripotency markers (OCT4, CDX2, and SOX2) of pXEN cells and pESCs. (**B**) AP staining and immunofluorescence staining of XEN markers (GATA4, GATA6, and SOX17) of pXEN cells and pESCs. Scale bar, 100μm.

Our lab also previously derived porcine ESCs in the primed state. The primed ESCs had a stable phenotype and a normally karyotype, and they expressed pluripotency genes as well as some lineage-associated genes and could form EBs in vitro [15,32]. We found that the pXEN cells had a similar morphology to that of primed-state ESCs, so we wondered whether pXEN cells might be molecularly identical to primed-state ES cells. Our comparison of the differences in the characteristics of the two types of cells revealed that both cell types had a flat morphology. The primed ES cells expressed pluripotent markers Oct4 and Sox2, and the trophoblast marker Cdx2; however, the pXEN cells did not express these markers (Figure 6A). The results of alkaline phosphatase (AP)staining showed that both XEN cells and primed ES cells were positive for AP, but the AP staining was stronger in the primed ESCs. The pXEN cells expressed the XEN markers Gata4, Gata6, and Sox17; however, the primed ES cells did not express these markers (Figure 6B). Therefore, although the two types of cells were similar in morphology, their characteristics and properties were completely different.

### 2.6. RNA-seq Analysis of Porcine XEN Cells, Naïve-like and Primed Embryonic Stem Cells

**Figure 7:**
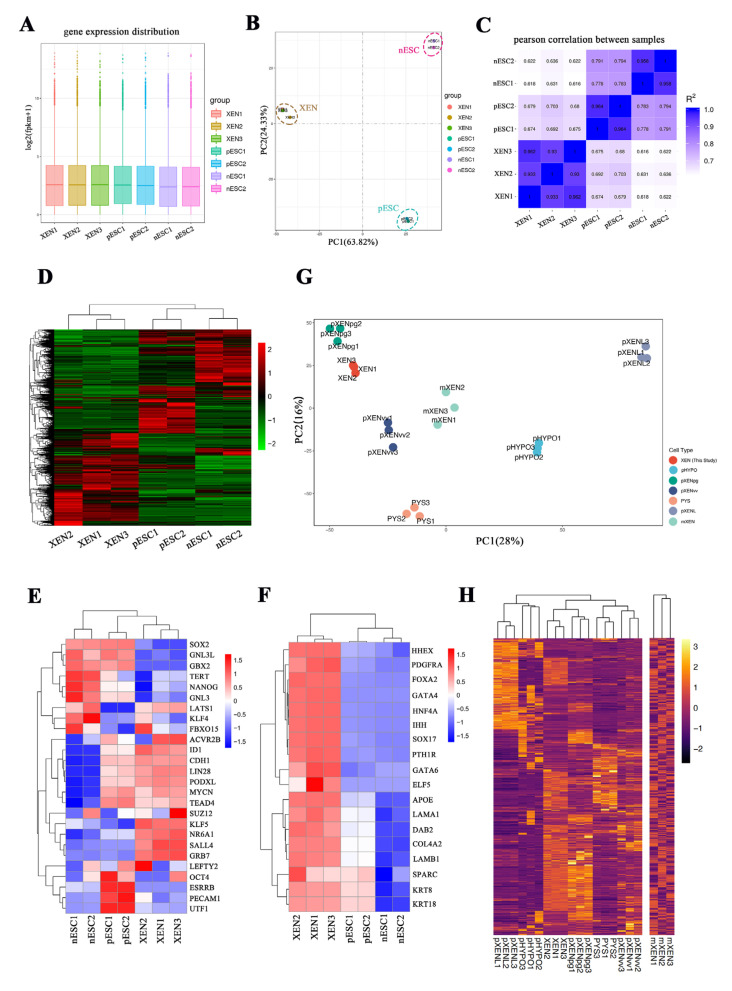
RNA-seq analysis of porcine XEN cells, nESCs and pESCs. (**A**) Gene expression distribution in porcine XEN cells, nESCs, and pESCs. (**B**) Principal component analysis (PCA) of global gene expression of porcine XEN cells, nESCs, and pESCs. (**C**) Pearson correlation analysis of different type of cells. (**D**) Hierarchical cluster of transcriptome data showing scaled expression values of 12,658 differentially expressed genes [log2 (fold change) >3, adjusted *p* value < 0.05] among the different type of cells. (**E**) Expression heatmap of pluripotency genes for each cell type. (**F**) Expression heatmap of XEN genes for each cell type. The results are shown by the Z score of the FPKMs of all samples for each gene. XEN1, XEN cell line 1 at passage 26; XEN2, XEN cell line 2 at passage 26; XEN3, XEN cell line 3 at passage 23; nESC1, porcine naïve-like ES cell line 1 at passage 21; nESC2, naïve-like ES cell line 2 at passage 22; pESC1, porcine primed ES cell line 1 at passage 20; pESC2, porcine primed ES cell line 2 at passage 20. (**G**–**H**) Transcriptome comparison of our XEN cells with analogous derivatives. RNA sequencing data analysis was performed on our XEN cells and published data from mouse XEN cells (the data for mXEN were from GSE106158 on NCBI), porcine XEN-like cells (data for pXENL were from GSE140414 [26]; data for pXENpg and pXENvv were from GSE128149 [27]), and porcine yolk sac (d 28, data for PYS were from GSE128149 [27]), as well as on porcine hypoblast cells from early embryos (data for pHYPO were retrieved from a previous study [35]). PCA plot (**G**) and heatmap (**H**) of global gene expression of these cells.

We determined the gene expression differences between pXEN cells and naïve-like and primed porcine ESCs using RNA sequencing analysis (Figure 7A). Global gene expression profiling revealed that gene expression in pXEN cells was distinct from that in porcine naïve like and primed ESCs (Figure 7B). Pearson correlation analysis confirmed that porcine naïve like and primed ESCs show a stronger correlation than the correlation between either pXEN cells and porcine naïve-like ESCs or primed ESCs (Figure 7C). Hierarchical cluster analysis illustrated the similarity between pXEN cell lines, which cluster together based on their gene expression patterns. The pXEN cells had a distinct identity and do not clustering with either porcine naïve-like ESCs or primed ESCs (Figure 7D). We selected genes related to pluripotency and typical XEN marker genes from RNA-seq data for analysis (Supplementary Table S1) and confirmed that pXEN cells lack the expression of key pluripotency genes, such as *Sox2*, *Oct4*, and *Klf4*, but they expressed other pluripotency genes, such as *Sall4*, *Lin28a*, and *Klf5* (Figure 7E). Oct4 is expressed in primitive endoderm of mouse blastocysts [33]. Previously known mouse blastocyst-derived XEN cell lines express XEN markers but not Oct4 [10]. The primitive XEN cells from mouse blastocysts have also been derived, which expressed oct4 and was easily convert into XEN-like cells but not vice versa [34]. The loss of Oct4 and Sox2 expression in our porcine XEN cells suggested that the status of these cells was more similar to that of mouse XEN cells than that of primitive XEN cells. The heterogeneity of pluripotency gene expression of pXEN cells (Figure 7E) may be due to the embryonic origin, since parthenogenetic embryos themselves are not normal naturally acquired embryos.

We also confirmed that pXEN cells robustly express *Gata4, Gata6*, and *Sox17*, as well as genes encoding ExEn-associated cell surface proteins or the basement membrane components *Pdgfra, Col4a2, Lama1, Lamb1, Sparc, Ihh, Hnf4a*, and *Dab2*. This expression pattern contrasts with that of porcine naïve-like ESCs, which express these genes at a low level or lack expression altogether (Figure 7F). We also compared our RNA sequencing data for pXEN cells with publicly available datasets from mouse XEN cells, porcine XEN-like cells, and porcine yolk sac (day 28), as well as porcine hypoblast cells of early embryos (Figure 7G). Hierarchical cluster analysis demonstrated that the pXEN cells derived in our lab clustered closely with the pXENpg cell lines (Figure 7H) derived by Park et al. [27]. We chose the signaling pathway-related genes as well (Supplementary Table S1) and analyzed the expression of them in these cell lines. The results showed that genes involved in the LIF signaling pathway were expressed at lower levels in pXEN cells (Supplementary Figure S2A), whereas genes related to the FGF and TGFβ signaling pathways showed higher expression in pXEN cells (Supplementary Figure S2C,D). The FGF and TGFβ signaling pathway-related genes were also expressed at high level in the primed ESCs (Supplementary Figure S2C,D). Surprisingly, we found that primed ESCs strongly expressed genes related to the WNT signaling pathway (Supplementary Figure S2B), while activation of this pathway is generally required for maintenance of naïve pluripotent stem cells in mammals. However, Choi et al. showed that FGF2, ACTVIN, and WNT signaling are essential to sustain pig pluripotency in vitro [13], and the porcine embryonic stem cells they obtained were in primed state. The gene Ctnnb1 encoding β-catenin1 was highly expressed in pXEN cells (Supplementary Figure S2B), and β-catenin1 is the cornerstone of the canonical WNT pathway. It is possibly due to the addition of CHIR99021 in the culture medium, since CHIR99021 is commonly used to activate WNT signaling in naive pluripotent stem cells.

## 3. Discussion

Understanding the development of pre-implantation embryos and their regulatory mechanisms is essential for understanding how to derive stem cells effectively. During blastocyst development, the ICM further differentiates to form the initial embryonic lineage epiblast-EPI and the extra-embryonic endoderm lineage PrE, and the epiblast stem cells (EpiSCs) and XEN cells, respectively, can be derived from these two lineages [23]. Over the past few decades, many researchers have tried and failed to obtain authentic ESCs with potency of germline chimerism from pigs [12,13,14].

The naïve-like and primed state of porcine stem cells was established by our group; however, we did not abandon the goal of deriving porcine ESCs with potency of germline chimerism and gamete development. In the present study, we cultured pig blastocysts using a chemical cocktail system that had proved capable of obtaining mouse and human extended pluripotent stem cells. Unexpectedly, we found that the extra-embryonic cells dominated when the pig blastocysts were cultured in this system, and that the derived cells were XEN cells rather than ES cells. This result suggested that the signaling pathways that maintain pluripotency programming in pig embryos likely differ from those in mice and humans. The culture system used for mouse and human ESCs therefore does not appear suitable for porcine cells. Species differences clearly exist, and the establishment of an authentic pig ESC line remains a great challenge.

In this study, we further confirmed the main reason why establishing porcine authentic ESCs has been so notoriously difficult. The epiblast fraction of the primary blastocyst outgrowths from pigs usually fails to proliferate under the routine ESC derivation conditions used for rodents and primates. Instead, the cells are rapidly replaced by expanding extra-embryonic cells, which are often mistaken for epiblast-derived primed ESCs, as found in previous studies [27,36]. Therefore, the discovery of ways to inhibit the growth of the PrE and to promote the proliferation of EPI may be significant for obtaining authentic porcine ESCs.

The findings presented here indicate the particular importance of studying the characteristics of porcine XEN cells and comparing them with different states of porcine ESCs. Previous studies had suggested Gata4 and Gata6 as master regulators of ExEn differentiation [20] and that the overexpression of Gata4 or Gata6 is sufficient to drive the establishment of self-renewing XEN cells from mESCs [37]. The role of the SOX factor Sox17 in ExEn development has also been revealed, as overexpression of Sox17 has been shown to induce ExEn gene expression in mESCs [22]; therefore, the commitment of cells to the ExEn fate can be measured by expression of these genes. The XEN cells we derived in this study robustly expressed Gata4, Gata6 and Sox17. We also found the presence of lipid droplets in pXEN cells, which was consistent with previous studies [27]. Other studies on pig XEN cells showed that some pXEN cells expressed AP [27], while others did not [26,28]; our pXEN cells expressed AP.

The LCDM system has also been used by Xu et al. to derive iPSCs from pigs [38]. For cell reprogramming, the basic medium used in the study was very different from ours, the basal medium might have a great influence on the cells. Notably, for the above iPSCs, the expression of exogenous transcriptional factors may play a key role during derivation of the pluripotent state. These differences in culture systems and with exogenous transcriptional factors transfected or not resulted in different stem cell types in our study and that of Xu et al. Besides, MIH is a chemical inhibitor of PARP1, and the upregulation of ExEm differentiation pathways observed in Parp1^−/−^ mouse ES cells [39] has indicated that PARP1 might be involved in the regulation of ExEm developmental potency. We speculate that the inhibition of PARP1 might be the main reason for the dominant growth of extra-embryonic cells of porcine blastocysts cultured in LCDM. However, the role of the PARP1 pathway in porcine embryonic development and pluripotent stem cells needs further study.

The fibroblast growth factor (FGF) receptor Fgfr2 is enriched in PrE cells, whereas both Fgf4 and Fgf2 are not expressed in early embryos [40]. Both factors function via Fgfr2 and are routinely added when XEN is obtained from embryos [10]. Our FGF/ERK signaling results showed that the proliferation of pXEN cells is blocked when ERK signaling, downstream of the FGF pathway, is inhibited. This is consistent with previous studies that demonstrated a requirement for the FGF/ERK signaling for the maintenance of pXEN cells [26]. Although the exogenous addition of FGF is not required for the derivation of pXEN cells in our study, the effect of exogenous production of FGF by the MEF feeder cells [41] should be taken into account. XEN cells can also be derived from ESCs by progressive adaptation of retinoic acid (RA) and activin A [24]. In addition, Nodal, a member of the TGFβ family, is expressed in PrE [42], suggesting that components of this pathway may also promote the derivation of XEN cells. Notably, in our analysis of signaling pathways, we found that maintenance of pXEN cells also required the TGFβ signaling pathway.

Mouse XEN cells express Lin28a, Sall4 and other pluripotency genes. Mouse fibroblasts pass through a XEN-like state on their way to the iPS cells state by chemical reprogramming [43]. Similar to XEN cells in vivo, the induced XEN-like cells have already expressed Sall4 and Lin28a, two master genes of pluripotency, and iPS cells can be rapidly obtained by changing culture conditions or by overexpressing Oct4 and Sox2 in XEN cells. The porcine XEN cells we derived also expressed Sall4 and Lin28a, as well as other pluripotency genes; therefore, porcine XEN cells may serve as a bridge to study cell fate transitions during the development of porcine embryos. Some studies have shown that cells in the chemically induced XEN-like state could be induced to form functional neuronal and hepatocyte-like cells [44], suggesting the possibility of generating other cell lineages via the XEN like state. Future studies on the induction of porcine XEN cells into other cell types will also be interesting.

Based on our observations and RNA sequencing results, we proposed that three types of cells could be derived from porcine ICMs: naïve-like ESCs, primed ESCs, and XEN cells (Figure S2E). The proliferation of porcine naïve-like ESCs depends on the LIF signaling pathway. By contrast, the growth of primed ESCs requires exogenous addition of FGF and upregulation of the expression of genes related to the TGFβ and WNT signaling pathways, which seems to indicate that they are dependent on FGF, TGFβ, and WNT signaling. In the present study, we demonstrated that the proliferation of XEN cells is dependent on the FGF and TGFβ signaling pathways. The conversion between porcine naïve-like and primed ESCs had be realized quickly via down-regulation of the LIF signaling pathway and up-regulation of the FGF [32]. Porcine naïve-like ESCs also can exit pluripotency and transition to the XEN state. The key pluripotency genes are downregulated, and ExEn-associated genes are upregulated in this process. These results suggest that distinct pluripotent stem cell states respond differently to differentiation-inducing signals. However, the further identification of signaling pathways in different cell types is needed.

In conclusion, successful production of pXEN cells using a chemical cocktail culture system represents an important intermediate step in the in vitro generation of cell lineages from porcine embryos. It also reveals important and novel molecular mechanisms of different cell types derived from porcine ICMs. The pXEN cells will be helpful for studying the lineage development of porcine embryos and for promoting the establishment of authentic porcine ESCs, which also represent a promising cell source for human regenerative medicine. The pXEN cells can serve as an in vitro tool to explore and identify the mechanisms and key molecules involved in cell reprogramming. Another interest of the pXEN cells could be the possibility of producing, together with nESCs and TSCs, porcine blastoïds to overcome the difficulty of obtaining embryos in vivo.

## 4. Materials and Methods

### 4.1. Porcine XEN Cell Lines Derivation and Maintenance

Porcine blastocysts were obtained by in vitro oocyte maturation (IVM) and parthenogenesis as previously reported [45]. Porcine expanded blastocysts at day 6 were selected to establish pXEN cells. The zona pellucida of blastocysts were removed by treatment in 0.5% pronase solution, and the ICMs were isolated by immunosurgery. The ICMs were seeded onto a monolayer of mitomycin C-inactivated feeder cells at a density of 5×10^4^ cell/cm^2^ and cultured with LCDM medium: knockout DMEM (Invitrogen, Carlsbad, CA, USA), 0.1 mmol β-mercaptoethanol (Invitrogen), 1% MEM non-essential amino acids (Invitrogen), 1% penicillin-streptomycin (Invitrogen), 1% Glutamax (Invitrogen), 10% KSR (Invitrogen), 10% FBS (BI, Kibbutz Beit-Haemek, Israel), 10 ng/mL hLIF (Millipore, Billerica, MA, USA), 1 μM CHIR99021 (Sigma, Darmstadt, Germany), 2 μM (*S*)-(+)-dimethindene maleate (DIM, Tocris, Ellisville, MO, USA), 2 μM minocycline hydrochloride (MIH, Tocris) and 2 μM Y27632 (Tocris). The feeder cells were mouse embryonic fibroblast (MEF) derived from ICR mouse strains and established in our lab. After 5-7 days, the outgrowths expanded from the edge of the embryos, and they were mechanically isolated from the feeder cells. The outgrowths were torn into small pieces and transferred onto fresh feeder cells for subculture. The pXEN cells were passaged as clumps every 5–6 days using accutase (Invitrogen) and were cultured in humidified conditions with 5% CO_2_ at 38.5 °C.

### 4.2. Culture Conditions for Different States of Porcine Pluripotent Stem Cells

Porcine naïve-like ESCs (nESCs) were established by our lab previously and maintained on MEF cells in LBX medium: knockout serum replacement (KOSR) medium and N2B27 medium supplemented with 16 ng/mL FGF (Invitrogen), 10 ng/mL hLIF (Millipore), 1 μM PD0325901 (Sigma), 3 μM CHIR99021(Sigma), 2 μM SB431542 (Tocris) and 2 mg/mL doxycycline (DOX, Tocris). Porcine primed ESCs (pESCs) were grown on STO cells in α-MEM (Invitrogen) medium supplemented with 20%KOSR, 0.1 mmol β-mercaptoethanol, 1%MEM non-essential amino acids, 1% insulin-transferrin-selenium (ITS) (Invitrogen), 10 ng/mL activin A (Invitrogen), 20 ng/mL bFGF (Invitrogen), 20 ng/mL epidermal growth factor (EGF, Invitrogen) and 10 ng/mL hLIF.

### 4.3. Alkaline Phosphatase Staining

Alkaline phosphatase (AP) activity was detected by using NBT/BCIP (Promega, Madison, WI, USA) in accordance with the manufacturer’s protocol. Cells were washed with phosphate buffered saline (PBS) (Invitrogen) and fixed with 4% paraformaldehyde (Solarbio, Beijing, China) in PBS for 10 min at room temperature. The fixed cells were washed once with PBS and incubated with the mixture at room temperature for 15 min. After the staining was stopped with PBS, the cells were observed and the images were captured.

### 4.4. Karyotyping

The cells were incubated in a medium supplemented with 0.02 μg/mL colcemid for 1–2 h at 38.5 °C in an atmosphere of 5% CO_2_. After trypsinization (Invitrogen) and treatment with hypotonic KCl (0.56%) (Sigma) for 30 min at 37 °C, the cells were fixed with a 3:1 (*v/v*) mixture of methanol and acetic acid and spread on clean microscopic slides by gentle dropping. After staining with Giemsa (1:10 dilution) (Sigma) for 20 min, the chromosomes were examined.

### 4.5. Immunofluorescence

For immunofluorescence staining, cells were washed with PBS and fixed with 4% paraformaldehyde for 10 min, permeabilized with 1%Triton X-100 (Sigma) for 1 h, blocked with 5%BSA (Sigma) for 1 h at room temperature, then incubated with primary antibodies at 4 °C overnight and then incubated with secondary antibodies for 1 h at room temperature. The primary antibodies included Gata4 (Santa Cruz Biotechnology, Dallas, TX, USA), Gata6 (Cell Signaling Technology, Danvers, MA, USA), Sox17 (Cell Signaling Technology), Sall4 (Santa Cruz Biotechnology), Oct4 (Santa Cruz Biotechnology), Sox2 (Calbiochem, Billerica, MA, USA), Nanog (Santa Cruz Biotechnology), Cdx2 (Bio Genex, San Ramon, CA, USA), Sparc (Santa Cruz Biotechnology), Cdh1 (Abcam, Waltham, MA, USA), Desmin (Millipore), Cytokeratin (Millipore), and βIII-Tubulin (Abcam). Secondary antibodies include goat anti-mouse IgG Alexa Fluor 546 (Thermo Fisher Scientific, Waltham, MA, USA), goat anti-mouse IgG Alexa Fluor 488 (Thermo Fisher Scientific), goat anti-rabbit IgG Alexa Fluor 546 (Thermo Fisher Scientific), and goat anti-rabbit IgG Alexa Fluor 488 (Thermo Fisher Scientific). The nuclei were stained with DAPI (Sigma).

### 4.6. Gene Expression Analysis

Total RNA was extracted using the Total RNA Kit (Promega) and was reverse transcribed into cDNA using HiScriptII Q RT Super Mix (Vazyme, Nanjing, China). Real-time PCR was carried out using ChamQ SYBR qPCR Master Mix (Vazyme) and performed on a 7500 Real-Time System (Applied Biosystems, Waltham, MA, USA). The primers used in this study are listed in Supplementary Table S2. The data were analyzed using the △△Ct method. All the results were normalized to β-actin expression.

### 4.7. Western Blotting and the Quatification of Protein Levels

Cells were lysed in RIPA buffer supplemented with protease inhibitor. Lysates were cleared by centrifugation for 15 min at 14,000× *g*. The protein content was determined by Pierce^TM^ BCA protein assay (Thermo), and 50 μg were resolved by electrophoresis on 10% SDS-polyacrylamide gels and transferred to PVDF membranes by electroblotting. Western blotting was performed using antibodies reactive with Gata4 (Santa Cruz Biotechnology, 1:500), Gata6 (Cell Signaling Technology, 1:1000), Sox17 (Cell Signaling Technology, 1:1000), Oct4 (Abcam, 1:1000), Sox2 (Cell Signaling Technology, 1:1000), Nanog (Abcam, 1:1000), Erk (Abcam,1:1000), pErk (Cell Signaling Technology,1:1000), Stat3 (Cell Signaling Technology, 1:1000), pStat3 (Cell Signaling Technology, 1:1000), Smad2/3 (Cell Signaling Technology, 1:1000), pSmad2/3 (Cell Signaling Technology, 1:1000) or Gapdh (Protein Tech, Wuhan, China; 1:3000), and reactivity was detected using anti-mouse IgG HRP-conjugated secondary antibody (Jackson Laboratories, West Grove, PA, USA; 1:3000) or anti-rabbit IgG HRP-conjugated secondary antibody (Abcam, 1:3000).

After western blotting, high-resolution images were acquired using the ChemiDoc XRS+ gel documentation system (Bio-Rad Laboratories, Hercules, CA, USA). For quantitative assessments, the samples were normalized by GAPDH expression and the amounts of other proteins were calculated by densitometric analysis of the bands using Image J software (version 1.8.0_112, National Institutes of Health, Bethesda, MD, USA).

### 4.8. Embryoid Body (EB) Formation Assay

For EBs formation, the XEN cells were digested into small clumps with accutase and cultured in knockout DMEM medium as hanging drops without LIF and small molecular inhibitors for 7 days. The EBs were subsequently placed on dishes coated with gelatin and cultured in the same conditions for an additional 5 days. The genes and markers associated with differentiation were detected by RT-PCR and immunocytochemistry, respectively.

### 4.9. VE and PE Differentiation

EBs were placed on a gelatin coated dish and allowed to grow adherently, VE and PE associated genes and markers were detected by RT-PCR and immunocytochemistry, respectively. The XEN cells were specifically differentiated into VE by passaging with accutase and seeding on Matrigel (Corning, New York, NY, USA) upon reaching confluence greater than 80%. The culture medium was changed to a knockout DMEM medium without LIF and small molecular inhibitors but supplemented with 10 ng/mL BMP4 and 1μg/mL heparin. The ability to differentiate into VE was evaluated after 6 days.

### 4.10. Signaling Pathway Analysis

The cells were cultured in medium supplemented with or without the JAK inhibitor SD1008 (Tocris), the FGF receptor (FGFR) inhibitor SU5402 (Tocris), Mek inhibitor PD0325901 (Sigma) and the TGFβ receptor inhibitor SB431542 (Tocris) for LIF, FGF and TGFβ signal pathway identification, respectively. Inhibitors were supplied at 1, 5 and 10 μM. After treatment for 5 days, the morphologic changes in the cells were evaluated, and the cells were incubated with 2 μM JC1 (Invitrogen) for 30 min.

### 4.11. RNA-seq Analysis of Global Gene Expression

The input materials for the RNA sample preparations were a total amount of 3 mg RNA per sample from the porcine XEN cell line 1 at passage 26, the porcine XEN cell line 2 at passage 26, the porcine XEN cell line 3 at passage 23, the porcine naïve-like ES cell line 1 at passage 21, the porcine naïve-like ES cell line 2 at passage 22, the porcine primed ES cell line 1 at passage 20 and the porcine primed ES cell line 2 at passage 20. The RNA-seq libraries were generated using the rRNA-depleted RNA with the NEBNext Ultra Directional RNA Library Prep Kit for Illumina (New England Biolabs, Ipswich, MA, USA), following the manufacturer’s recommendations. The RNA-seq libraries were sequenced on an Illumina NovaSeq 6000 platform. The top 90% expressed genes [log2 (normalized counts)] in the whole RNA-seq data set were used for clustering analysis. Transcripts with a *p* adjust <0.05 were assigned as differentially expressed genes (DEGs), and gene expression levels were quantified with the fragments per kilobase million (FPKM).

### 4.12. Transcriptomics Data Availability

RNA sequencing datasets used in this study have been deposited in the National Center for Biotechnology Information database (http://www.ncbi.nlm.nih.gov/geo, accessed on 1 December 2021) under the accession number GSE183270. The data for mouse XEN cells and porcine XEN-like cells were downloaded from GSE106158 and GSE140414 [26], respectively. The data for porcine XEN cells and yolk sac were downloaded from GSE128149 [27]. Data for porcine hypoblast cells were retrieved from a previous study [33].

### 4.13. Statistical Analysis

All values are depicted as mean ± standard deviations (SD). Statistical parameters, including statistical analysis and statistical significance were reported in the figure legends. Statistical analyses were performed using Prism Software (GraphPad Software, San Diego, CA, USA). For statistical comparison, the student *t*-test was performed when two value sets were compared. A value of P< 0.05 was considered statistically significant.

## Data Availability

Not applicable.

## Supplementary Materials

The following are available online at https://www.mdpi.com/article/10.3390/ijms222312918/s1.

## Abbreviations

ESCs	Embryonic stem cells
pXEN	Porcine extra-embryonic endoderm
VE	Visceral endoderm
PE	Parietal endoderm
ICM	Inner cell mass
TE	Trophectoderm
PrE	Primitive endoderm
EPI	Epiblast
ExEn	Extra-embryonic endoderm
nESCs	Naïve-like ESCs
pESCs	Primed ESCs
AP	Alkaline phosphatase
EB	Embryoid body
KOSR	Knockout serum replacement
