# Supplementary material for: Derivation of Porcine Extra-Embryonic Endoderm Cell Lines Reveals Distinct Signaling Pathway and Multipotency States
Source: Int J Mol Sci. 2021 Nov 29;22(23):12918. doi: 10.3390/ijms222312918 (PMC8657774; doi:10.3390/ijms222312918)
Supplement: Supplementary file 1 [file ijms-22-12918-s001.zip › ijms-1468859-supplementary.pdf]

Supplementary Materials

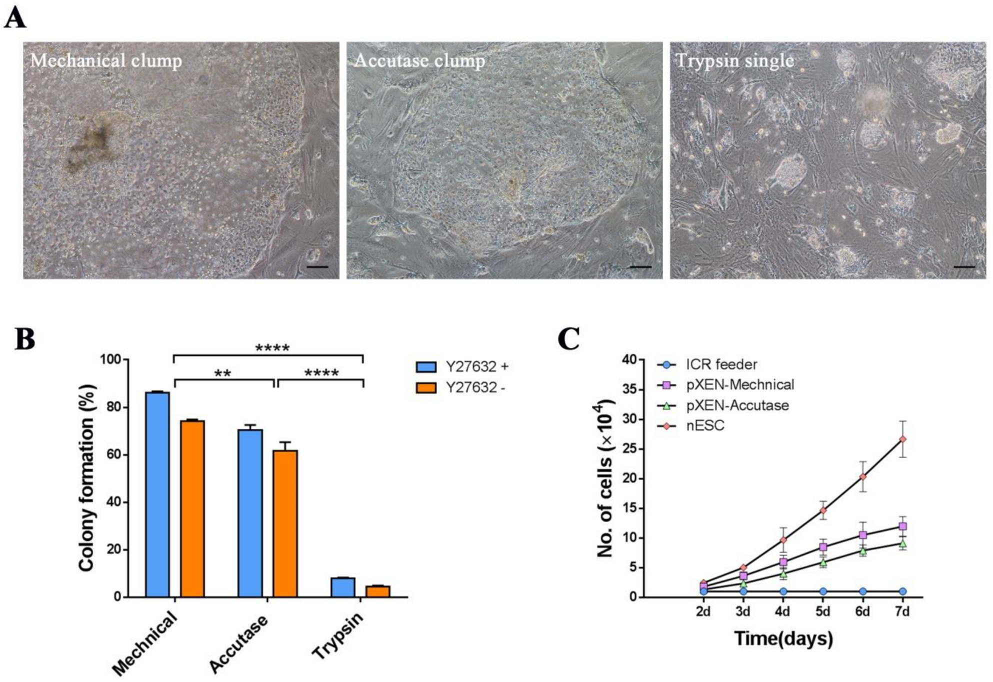

Supplementary Figure S1. Colony formation efficiency and proliferation ability of pXEN cells. A. Bright field images showed the morphology of colonies cultured for 6 days after passaged through different methods. B. Colony formation efficiency of pXEN cells passaged through mechanically method or accutase into clumps for passage, or digested into single cells by trypsin. C. Cell proliferation of pXEN after 2 days of passage. The ICR mouse derived MEF (ICR feeder) and porcine naïve-like ESCs (nESC) established in our lab were used as control.

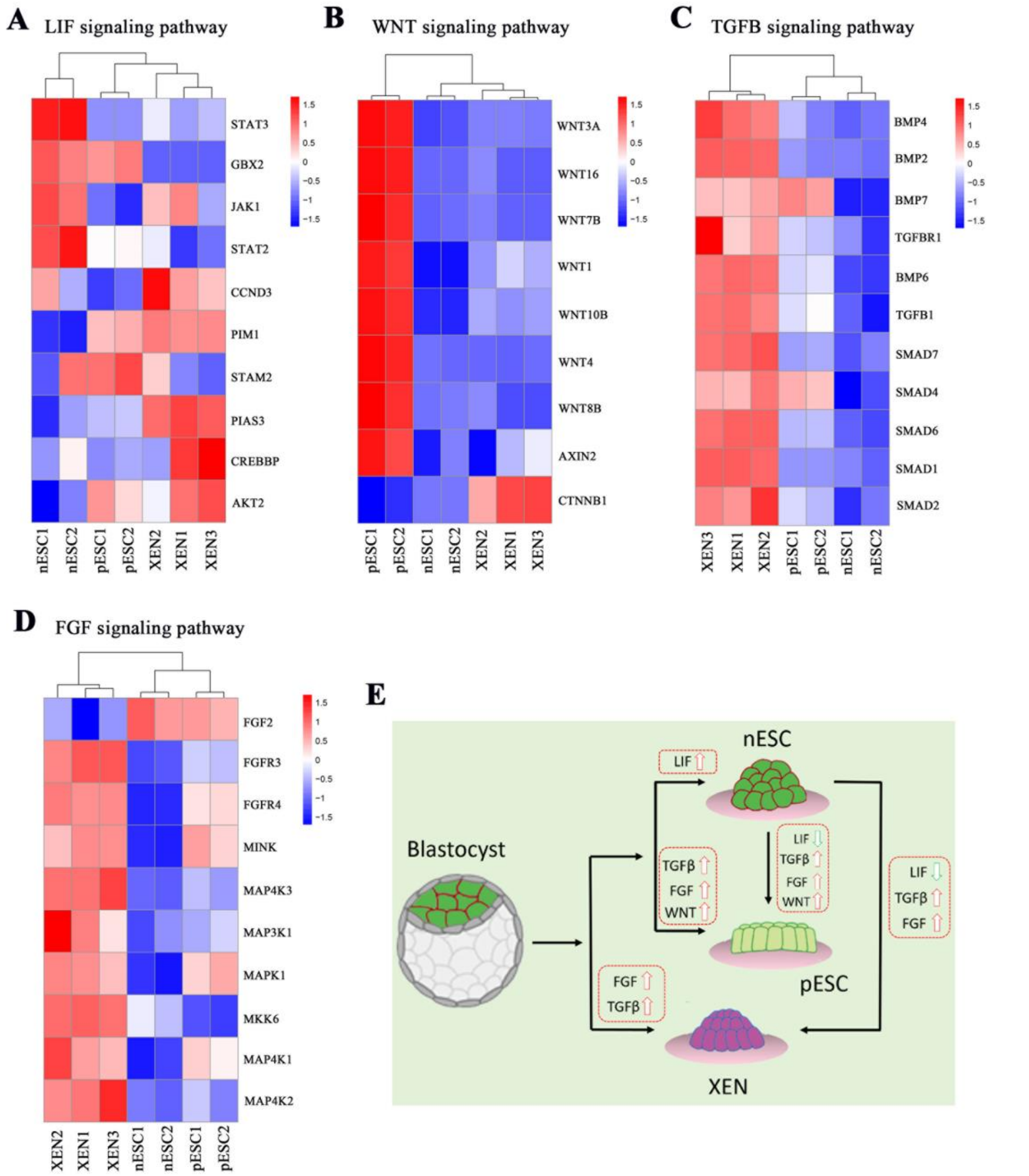

Supplementary Figure S2. Analysis of gene expression in porcine XEN cells, nESCs and pESCs from RNA-seq data. A. Expression of LIF signaling related genes in each cell type. B. Expression of WNT signaling related genes in each cell type. C. Expression of TGFβ signaling related genes in each cell type. D. Expression of FGF signaling related genes in each cell type. E. A schematic model of different cell types derivation from porcine ICMs and the conversion between them, and the distinct stem cell states might respond to different signaling pathway.

Supplementary Table S1. The Normalized Data of mRNA Analysis

gene_id	XEN1	XEN2	XEN3	pESC1	pESC2	nESC1	nESC2	gene_name	gene_biotype
ENSSSCG00000007371	276.508339	211.2657712	268.0953247	0.217429878	0.14094041	0	0.018062571	HNF4A	protein_coding
ENSSSCG00000022383	238.9421955	187.9614749	239.0582171	0.038623379	0	0	0.083422645	GATA4	protein_coding
ENSSSCG00000003702	96.62514315	49.15395827	90.23606561	8.083069448	9.841097478	12.09054445	12.10390202	GATA6	protein_coding
ENSSSCG00000006256	333.8320624	262.7335919	314.523391	0.790273979	0.317115923	0	0	SOX17	protein_coding
ENSSSCG00000008841	22.33873405	13.9315068	24.73880862	0.542483456	0.39249117	0.025654597	0	PDGFRA	Processed transcrit
ENSSSCG00000009545	857.1995023	813.3778659	793.3807969	69.44883838	69.33823126	0.805814972	0.733724138	COL4A2	protein_coding
ENSSSCG00000016857	42.45510466	47.853716	28.0184485	7.495762313	8.463264194	0.589810808	0.680256253	DAB2	protein_coding
ENSSSCG00000007114	97.81607281	103.5385289	99.57047187	0.110686756	0.103636518	0	0	FOXA2	protein_coding
ENSSSCG00000017083	115.3370239	161.9187743	112.32182	109.8941155	118.2417731	50.71629713	77.88866746	SPARC	protein_coding
ENSSSCG00000011326	109.2797831	81.7751665	104.6833708	0.386422285	0.470351647	0.160813879	0.125195038	PTH1R	protein_coding
ENSSSCG00000000253	2757.599351	3349.433719	2542.807775	707.8843537	794.7129935	0.11684668	0.545797092	KRT18	protein_coding
ENSSSCG00000000252	7715.138869	9097.869643	6963.187279	1892.301475	2239.309067	5.302200145	18.76277304	KRT8	protein_coding
ENSSSCG00000015444	307.2713298	344.0398785	286.6614524	74.16099798	80.34850343	8.137682397	12.77006045	LAMB1	protein_coding
ENSSSCG00000028866	153.0022697	111.7840672	111.5153481	35.42842092	34.60573225	5.523661244	8.655570039	LAMA1	protein_coding
ENSSSCG00000016204	101.6954963	86.45286279	108.4875632	0.155494749	0.029118089	0	0	IHH	protein_coding
ENSSSCG00000003088	8585.40263	7972.647807	8928.407911	45.91170136	62.09281961	1.046530611	1.241498527	APOE	protein_coding
ENSSSCG00000013301	1.09801299	0.282020353	0.590183322	0	0	0	0	ELF5	protein_coding
ENSSSCG00000010472	122.0689144	115.7857997	122.9269328	5.849556559	5.124875934	3.303770514	1.218322795	HHEX	protein_coding
ENSSSCG00000003557	625.319464	534.1045643	611.8042778	401.783134	450.9258426	121.0327408	118.1618321	LIN28	protein_coding
ENSSSCG00000011771	0	1.977138132	0	147.3021815	153.5003607	102.0648659	125.8618584	SOX2	protein_coding
ENSSSCG00000002388	0	0	0	36.64432952	37.70043372	1.180054902	0.800903354	ESRRB	protein_coding
ENSSSCG00000001393	0	0	0.195458563	0.28379185	0.159429367	0	0.122592417	POU5F1	protein_coding
ENSSSCG00000005437	0.441526144	0.675025401	0.282524811	0.344572884	0.368714483	0.921842941	1.19610491	KLF4	protein_coding
ENSSSCG00000004977	1.1942044	0.4967525	1.0176415	1.4346302	1.4072152	2.4967525	2.1475552	NANOG	protein_coding
ENSSSCG00000007479	115.7360494	90.38254023	112.1360095	39.77074516	39.60557769	33.7796311	37.72672117	SALL4	protein_coding

ENSSSCG00000008618	80.42976242	57.8425979	74.86527802	70.86857804	80.9072441	14.02490985	15.81692088	MYCN	protein_coding
ENSSSCG00000017118	12.23565359	8.520822657	12.08871171	14.32330057	11.48595214	19.89309673	19.07035404	TERT	protein_coding
ENSSSCG00000011449	55.35910001	44.4506445	51.10981415	69.0999847	69.95805537	96.51462639	82.29663502	GNL3	protein_coding
ENSSSCG00000016319	0	0	0	17.46905537	23.72025755	34.93347452	21.96915832	GBX2	protein_coding
ENSSSCG00000000736	25.22809954	24.45184505	28.50917722	31.33684013	33.53947225	8.392285688	11.89206018	TEAD4	protein_coding
ENSSSCG00000017744	27.71896745	28.45671867	30.48194272	27.00793068	28.28942076	27.15574195	29.30710872	SUZ12	protein_coding
ENSSSCG00000005589	86.09876166	77.74049774	94.92961944	35.73119284	36.751712	16.44420805	21.6804168	NR6A1	protein_coding
ENSSSCG00000016548	317.652361	269.3038777	286.8515102	178.8807465	181.5652375	20.00600257	24.60819906	PODXL	protein_coding
ENSSSCG00000029429	0	0.314718365	0.082326297	0.071719136	0.201452869	0	0.154906179	LEFTY2	protein_coding
ENSSSCG00000017277	0.11704707	0.200420555	0.146796867	0.310573315	0.342107317	0.133049923	0.138107401	PECAM1	protein_coding
ENSSSCG00000011257	50.99178551	23.34654267	51.94839882	41.36551085	34.07322626	21.12926872	29.84911098	ACVR2B	protein_coding
ENSSSCG00000012336	19.14803003	17.64728854	17.50664246	35.82015538	33.99124374	37.9075537	30.68612735	GNL3L	protein_coding
ENSSSCG00000010771	0.381056903	1.712777128	0.256023188	135.9035204	142.2132409	0	0.0802894	UTF1	protein_coding
ENSSSCG00000025652	102.2609004	123.2143194	111.0678214	66.41179368	62.6388853	0.798366524	1.205402094	CDH1	protein_coding
ENSSSCG00000004873	1.987949061	3.282409031	1.717272397	1.496014003	1.141331298	3.401146019	2.213998466	FBXO15	protein_coding
ENSSSCG00000004106	11.47488164	10.962297	11.64049812	8.256521725	8.265815889	11.18916311	12.41469481	LATS1	protein_coding
ENSSSCG00000028760	19.48500403	23.77686823	19.66235475	1.048714255	0.327305323	4.182492095	2.45387802	KLF5	protein_coding
ENSSSCG00000007227	356.7174634	440.6396321	316.1132608	209.4817191	201.465736	52.28714017	54.53068547	ID1	protein_coding
ENSSSCG00000017495	27.57690356	19.62395176	31.57942088	0.279498479	0	0.083083116	0	GRB7	protein_coding
ENSSSCG00000016319	0	0	0	17.46905537	23.72025755	34.93347452	21.96915832	GBX2	protein_coding
ENSSSCG00000017403	14.46450295	18.80378674	16.04125898	13.68665285	13.65323223	42.18655948	44.27363753	STAT3	protein_coding
ENSSSCG00000003809	7.729165348	6.465230151	3.979344644	3.327968881	2.531743933	9.377406513	8.311218409	JAK1	protein_coding
ENSSSCG00000001573	42.12200531	51.66036563	44.66447984	28.40504332	32.36148714	1.758714042	1.336575163	PIM1	protein_coding
ENSSSCG00000000396	2.37602811	5.318098078	3.071164737	5.774770134	5.853388673	11.46496968	14.1893853	STAT2	protein_coding
ENSSSCG00000026752	12.79652573	14.47351292	12.41703092	9.986579138	10.39946988	12.74139074	11.03533633	CCND3	protein_coding
ENSSSCG00000016399	16.56951261	18.33274011	16.21372977	19.39660254	19.88410677	16.14421845	19.3897388	STAM2	protein_coding
ENSSSCG00000006689	14.43334929	13.26245164	13.75874481	8.623338506	8.846709825	6.267341735	8.065130898	PIAS3	protein_coding
ENSSSCG00000007951	11.27293337	8.763200923	11.8644147	8.590652079	8.843602417	8.694404561	9.617487947	CREBBP	protein_coding
ENSSSCG00000002989	61.87186	56.30806	63.16217	60.40626	58.17696	48.55207	52.64225	AKT2	protein_coding
ENSSSCG00000009086	3.835121661	8.059385748	7.495936632	17.14161291	15.54025429	21.79732657	17.04287367	FGF2	protein_coding

ENSSSCG00000030827	82.84796963	64.00377687	83.06053707	22.22538282	19.68647555	9.131092842	10.36463164	FGFR3	protein_coding
ENSSSCG00000014047	31.76396131	38.47834707	33.78724445	13.31876521	15.17752305	0.320532722	0.38390397	FGFR4	protein_coding
ENSSSCG00000017917	29.52759284	26.30798317	29.44943458	28.26643995	24.99151661	13.37691507	12.95970368	MINK	protein_coding
ENSSSCG00000008475	14.71977604	14.60463906	17.19174009	6.978393503	6.081554806	4.970538947	4.811648484	MAP4K3	protein_coding
ENSSSCG00000016918	8.733984411	11.25371305	7.200354689	5.70651721	6.21234209	4.653064387	5.392409704	MAP3K1	protein_coding
ENSSSCG00000010081	130.5395	133.5609	121.0346	116.4407	125.4305	75.41038	70.85771	MAPK1	protein_coding
ENSSSCG00000017254	5.147108	4.951876	4.801104	1.076687	0.930561	2.455797	1.967762	MKK6	protein_coding
ENSSSCG00000002962	2.039905	3.163421	1.792939	1.622006	1.321824	0.031251	0.194632	MAP4K1	protein_coding
ENSSSCG00000013020	2.325915	2.090907	3.417164	0.671615	0.288925	0.264392	0.156824	MAP4K2	protein_coding
ENSSSCG00000005045	27.12267768	22.75791438	39.20312392	4.803460861	2.718270709	1.922127889	2.451235382	BMP4	protein_coding
ENSSSCG00000027638	30.40197529	29.22002836	32.60915315	0.590880047	0.170228831	0.14186609	0	BMP2	protein_coding
ENSSSCG00000007501	13.74193318	18.00053824	14.04874422	24.26989095	19.07263083	0	0.022401942	BMP7	protein_coding
ENSSSCG00000001027	51.58352	48.81379	48.5485	25.28244	26.75188	15.09217	14.1498	BMP6	protein_coding
ENSSSCG00000023995	7.094853169	8.149665062	13.00294923	5.398494974	5.141039643	4.392475071	3.189117427	TGFBR1	protein_coding
ENSSSCG00000003017	35.92707482	29.73823197	34.73908906	7.606259653	10.87207265	2.188860141	0.699096258	TGFB1	protein_coding
ENSSSCG00000004507	20.97272833	24.66809128	20.03275011	3.64006769	4.060848437	1.853279021	2.815888266	SMAD7	protein_coding
ENSSSCG00000004948	97.13910668	100.0570813	85.76143383	8.031407664	8.436897976	2.40486772	1.565464526	SMAD6	protein_coding
ENSSSCG00000009040	86.07359404	80.67122139	87.35477989	8.413723484	8.422209265	7.045627683	5.023376544	SMAD1	protein_coding
ENSSSCG00000004952	30.88562591	47.54928179	33.86216168	16.25525143	14.18649753	7.343183468	10.47699987	SMAD2	protein_coding
ENSSSCG00000004524	46.82827	49.78509	47.03453	47.28871	46.44581	35.06808	37.65207	SMAD4	protein_coding
ENSSSCG00000029870	0.672532957	0.658047489	0.602478808	33.81557268	28.99395152	0	0.161947369	WNT3A	protein_coding
ENSSSCG00000016617	0	0.39881666	0	21.01232387	17.97203774	0.075644545	0.078519937	WNT16	protein_coding
ENSSSCG00000027365	0	0.212507079	0	16.34406735	12.10639332	0	0.0522986	WNT7B	protein_coding
ENSSSCG00000000183	0.729246363	0.468260208	0.571624098	2.774428684	2.464494394	0	0	WNT1	protein_coding
ENSSSCG00000003521	0	0.078679591	0.109768396	51.04011854	37.00017689	0.174105787	0.07745309	WNT4	protein_coding
ENSSSCG00000006164	0.510858008	1.399593452	0.549173605	39.5490951	26.87661234	0.995491354	1.205553831	WNT8B	protein_coding
ENSSSCG00000000182	1.976423	2.608688	2.360733	56.94127	44.34841	0.200593	0.138812	WNT10B	protein_coding
ENSSSCG00000017265	6.047888	2.007765	7.961013	29.0897	22.36666	2.30495	4.369033	AXIN2	protein_coding
ENSSSCG00000011274	486.8679	421.6171	488.5964	255.4057	274.5786	305.1061	305.2941	CTNNB1	protein_coding

Supplementary Table S2. List of primers and conditions for PCR

Gene ID	Symbol (species)	Primer sequence（5′-3′）	Annealing Temperature (℃)	Products (size bp)
397555	GATA4 (pig)	F-ATGCGTCCCATCAAGACAGAGC<br>R-GGTGACTGGCTGACCGAAGAT	58	173
397600	GATA6 (pig)	F- CTGGTGTCTGCCAAAGCG<br>R- AAGGGATGCGAGGCGTAA	60	243
100154087	SOX17 (pig)	F- CTGGAGGAGCGGAGCAAATC<br>R- CAGGGCAACTGTGGGAAACC	58	173
100136902	SALL4 (pig)	F-TTAGACCCGTCCAGGAAAGGC<br>R-GCAAGGAGCTATCAGTCCCAAAA	56	133
100127461	OCT4 (pig)	F- GTCGCCAGAAGGGCAAAC<br>R-GTGACAGACACCGAGGGAAA	56	211
407739	SOX2 (pig)	F-TCGCAGACCTACATGAACGG<br>R-CCCTGGAGTGGGAAGAAGAG	57	151
100170132	NANOG (pig)	F- AGCGAATCTTCACCAATG<br>R- CTTCTGCCTCTGAAATCTG	54	297
100519746	DAB2 (pig)	F- GGACTGGCAAACAGGAAGCG<br>R- TTGTGGAGGTGGGATAATGG	55	248
100625589	LAMA1 (pig)	F-TGGCAAATCAGAAGAGGAGTC<br>R-CAGAGGGTCACAGTCACAAGG	54	182
100515336	COL4A1 (pig)	F-CCTCTGGATTGGCTACTCCTTTG<br>R- TGAACATCTCGCTCCGCTCTAT	58	200
100155811	SOX7 (pig)	F-ACAGCCGTCCTTGACTTTCG<br>R-CCAATCCGTCCCTCACTTTA	55	137
733636	HNF4A (pig)	F-CCACAGGCAAACACTACGGG<br>R-TTCTGGACGGCTTCCTTCTTC	57	196
100627123	PDGFRA (pig)	F-TGGACAAGTGAAAGGCAAAG<br>R- CTCGTGGACAGAAATGGTGA	54	184
100521224	NEFL (pig)	F-AAACGCCGCTATGTGGAGAC<br>R-CGAGACTGGGCATCAAGGAG	59	163
100153933	NESTIN (pig)	F-CCACTCCTCTGGGCTTCTACC<br>R-CCTCATCTCCCTCCTCCTCTG	60	168
733615	ACTA2 (pig)	F-TGGTTCTGGGCTCTGTAAGG<br>R-GATGCCGTGTTCTATTGGGT	55	190
100627924	MYH11 (pig)	F-AAGTTCAAGTCCACCATTTCGG<br>R-CCCTCGCATTTCCTTTCTCC	57	205
396960	ALBUMIN(pig)	F-GCGCTCATAGTTCGTTACAC<br>R-TCAGGACCAGGGACAGATAG	52	157
100156098	NCSTN (pig)	F- CCCAGGATGACAGATAGAAGC<br>R-ACAGGGCAGCACAGAAAGAG	53	101
595124	SPARC (pig)	F-GGCATCAAGGAGCAGGACAT<br>R-CAGGCAGAACGACAAACCAT	54	144

396583	SNAIL1 (pig)	F-CTCACTGCCCACGCTCATCT<br>R-GCCTTTCCCACTGTCCTCGT	58	136
397586	AFP (pig)	F-CAAAGGCAGCATCAATCACA<br>R-GCCACATCCAGGACCAGTTT	54	178
100513828	FOXA2 (pig)	F-CCCCAACAAGATGCTGACCC<br>R-AGCGAGTGGCGGATGGAGTT	56	108
397576	APOE (pig)	F-GAAGATGAGGGTTCTGTGGGTT<br>R- TGGGTGACCTTGGTGCTGA	59	228
397691	APOA1 (pig)	F-ACCTTGGCTGTGCTCTTCC<br>R-CAGGCTGTCCCAGTTGTCC	55	210
100153243	APOA2 (pig)	F- CAAGAAGGCTGGAACTGATA<br>R-ACAAGGAGTGGGTAGGGAGA	52	165
396916	H19 (pig)	F-CCGTGGCATCGTGGAAGA<br>R-CCAGGTGTCATAGCGGAAGAA	59	169
100048953	CDH1 (pig)	F-CACAGACCCAGTAACCAATG<br>R- AATCCTCAGGCACTTCCAC	53	218
100302020	CLDN6 (pig)	F-CCTTCATCGGCAACAGCATC<br>R-GCCAGCAGCGAGTCATACACC	57	115
100217393	LGALS2 (pig)	F-TGATGACGCTGATGGCTTTG<br>R-TGTGACTGTCTCGTTGCTCCTT	55	146
100156435	TNNC1 (pig)	F-GCAGGAGATGATTGACGAGG<br>R-ATGCGGAAGAGGTCAGAAAG	53	138
397643	BFGF (pig)	F-AGCACTCCCACCACTACAA<br>R-GAGCCAACGCCTAACAAC	57	156
100153248	FGFR1 (pig)	F-GCGGGTAACTCTATCGGACTC<br>R-TTGGTGCCACTCTTCATCTTG	56	185
445013	ERK1 (pig)	F-GCCTCAAATACATCCACTCCG<br>R-GCAGCCCACAGACCAAAT	55	242
396762	FGFR2 (pig)	F-CTGCCGCCAACACTGTCA<br>R-CGGATGGAACCACGCTTT	54	165
397078	TGFβ (pig)	F-CTGAGCATCTTGGACCTTATC<br>R-CACTGAGCCACAATGGAAAC	58	136
396665	TGFβR (pig)	F-ATGATTCAGCCACAGATAC<br>R-GCATAGATGTCAGCACGT	56	136
100155304	SMAD2 (pig)	F-GTCCATCTTGCCATTCACTC<br>R-GCTCATCTAATCGTCCTGTTT	52	176
397260	SMAD3 (pig)	F-CCACCAGATGAACCACAGCA<br>R-GGAGATGGAGCACCAGAAGG	56	130
110256843	NODAL (pig)	F-GTCACATAGCCATCTAATCCAA<br>R-CAGACTCCACAGACCCTTCAT	54	129
397451	LIFR (pig)	F- GGGTCAATCAGAATCAACAC<br>R-GGTAAATGCCAGGAAAGAGT	56	120
100037294	GP130 (pig)	F-CAGTAGTGGTATTGGAGGGT<br>R-CTAACAAGGGCTGGGTGG	52	191

733648	STAT3 (pig)	F-AGAAGGACATCAGCGGTAAGA<br>R-GAGGTAGACCAGCGGAGACA	53	146
414396	β-actin (pig)	F-GATGGCGTAACCCACAAC<br>R-AGGGCAACATAGCACAGC	53	194
